# Development and Feasibility Study of HOPE Model for Prediction of Depression Among Older Adults Using Wi-Fi-based Motion Sensor Data: Machine Learning Study

**DOI:** 10.2196/67715

**Published:** 2025-03-03

**Authors:** Shayan Nejadshamsi, Vania Karami, Negar Ghourchian, Narges Armanfard, Howard Bergman, Roland Grad, Machelle Wilchesky, Vladimir Khanassov, Isabelle Vedel, Samira Abbasgholizadeh Rahimi

**Affiliations:** 1 Mila—Quebec Artificial Intelligence Institute Montreal, QC Canada; 2 Family Medicine Department Faculty of Medicine and Health Sciences McGill University Montreal, QC Canada; 3 Lady Davis Institute for Medical Research Jewish General Hospital Montreal, QC Canada; 4 Aerial Technologies Montreal, QC Canada; 5 Department of Electrical and Computer Engineering Faculty of Engineering McGill University Montreal, QC Canada; 6 Donald Berman Maimonides Centre for Research in Aging Montreal, QC Canada; 7 Faculty of Dental Medicine and Oral Health Sciences McGill University Montreal Canada

**Keywords:** depression, classification, machine learning, artificial intelligence, older adults

## Abstract

**Background:**

Depression, characterized by persistent sadness and loss of interest in daily activities, greatly reduces quality of life. Early detection is vital for effective treatment and intervention. While many studies use wearable devices to classify depression based on physical activity, these often rely on intrusive methods. Additionally, most depression classification studies involve large participant groups and use single-stage classifiers without explainability.

**Objective:**

This study aims to assess the feasibility of classifying depression using nonintrusive Wi-Fi–based motion sensor data using a novel machine learning model on a limited number of participants. We also conduct an explainability analysis to interpret the model’s predictions and identify key features associated with depression classification.

**Methods:**

In this study, we recruited adults aged 65 years and older through web-based and in-person methods, supported by a McGill University health care facility directory. Participants provided consent, and we collected 6 months of activity and sleep data via nonintrusive Wi-Fi–based sensors, along with Edmonton Frailty Scale and Geriatric Depression Scale data. For depression classification, we proposed a HOPE (Home-Based Older Adults’ Depression Prediction) machine learning model with feature selection, dimensionality reduction, and classification stages, evaluating various model combinations using accuracy, sensitivity, precision, and *F*_1_-score. Shapely addictive explanations and local interpretable model-agnostic explanations were used to explain the model’s predictions.

**Results:**

A total of 6 participants were enrolled in this study; however, 2 participants withdrew later due to internet connectivity issues. Among the 4 remaining participants, 3 participants were classified as not having depression, while 1 participant was identified as having depression. The most accurate classification model, which combined sequential forward selection for feature selection, principal component analysis for dimensionality reduction, and a decision tree for classification, achieved an accuracy of 87.5%, sensitivity of 90%, and precision of 88.3%, effectively distinguishing individuals with and those without depression. The explainability analysis revealed that the most influential features in depression classification, in order of importance, were “average sleep duration,” “total number of sleep interruptions,” “percentage of nights with sleep interruptions,” “average duration of sleep interruptions,” and “Edmonton Frailty Scale.”

**Conclusions:**

The findings from this preliminary study demonstrate the feasibility of using Wi-Fi–based motion sensors for depression classification and highlight the effectiveness of our proposed HOPE machine learning model, even with a small sample size. These results suggest the potential for further research with a larger cohort for more comprehensive validation. Additionally, the nonintrusive data collection method and model architecture proposed in this study offer promising applications in remote health monitoring, particularly for older adults who may face challenges in using wearable devices. Furthermore, the importance of sleep patterns identified in our explainability analysis aligns with findings from previous research, emphasizing the need for more in-depth studies on the role of sleep in mental health, as suggested in the explainable machine learning study.

## Introduction

Depression is a prevalent mental health disorder characterized by emotional dysregulation, leading to persistent sadness, loss of interest, and anhedonia [1-3]. The rising incidence of depression among older adults has become a significant public health issue [4-6]. Early detection of depression and corresponding intervention are vital for improving mental health outcomes and reducing the overall burden on individuals and health care systems [7-9]. Traditional methods for assessing depression include various approaches that typically require in-person evaluations, specialized training in comprehensive geriatric assessments, and reliance on clinical judgment and questionnaires, which can be challenging and resource-intensive [10-12]. These methods require older adults to visit clinical settings frequently, increasing strain on health care facilities and reducing data collection opportunities. Additionally, many older adults prefer to remain in their homes and be remotely monitored in that environment, highlighting the need for remote care solutions in this demographic [13,14].

Physical activity and mobility are among the important factors in evaluating depression, with strong correlations established between these parameters and depression assessments [1,15]. The advent of the Internet of Things has enabled continuous and remote monitoring of physical activity. Several studies have used statistical methods to analyze the relationship between physical activity, as measured by wearable devices, and depression [16-21]. As the field of artificial intelligence (AI) advances, machine learning models have emerged as promising tools for depression classification using physical activity data [22]. For instance, Adamczyk and Malawski [23] used data from wearable actigraph watches in 3 classification models: logistic regression (LR), support vector machine (SVM), and random forest (RF) comparing automatic and manual feature engineering for depression classification. Bai et al [24] used phone use, sleep data, and step counts from 334 participants, using 2 feature selection methods (L1-based feature selection) and 6 machine learning models (decision tree [DT], k-nearest neighbors, naive Bayes, LR, SVM, and RF) for mood classification. Chikersal et al [25] analyzed data from smartphones and fitness trackers of 138 college students to identify those experiencing depressive symptoms, using nested randomized LR for feature selection and AdaBoost with gradient boosting classifier. Dai et al [26] used heart rate, energy expenditure, sleep, and other activity data from wearable Fitbit devices for depression remission detection in 106 participants within 2 intervention and control groups, using a multitask learning algorithm comprising 2 dense layers with shared parameters. Similarly, Griffiths et al [27] classified depression using activity and sleep data from Fitbit devices of 24 participants through an RF model. Espino-Salinas et al [28] used wrist-worn accelerometers to measure physical activity in 55 participants, applying a 2D-convolutional neural network (CNN) and a deep neural network for depression classification. Jakobsen et al [29] used RF, deep neural network, and CNN algorithms for depression classification with wrist-worn actigraph data from 55 participants. Jung et al [30] used gait accelerometry data and a bidirectional long short-term memory network–based classifier to assess depression in 45 older adults. Other studies have also explored the use of wearable device data combined with various classification methods, such as 1D-CNN [31], deep convolutional neuro-fuzzy [32], Ensemble models [33], and extreme gradient boosting [34].

These recent studies highlight the integration of wearable devices with machine learning algorithms as a promising approach for continuous, remote, home-based monitoring and early detection of depression. However, wearable device-based approaches face challenges due to their intrusive nature [35]. Participants are required to wear sensors or devices, which may lead to issues with compliance, comfort, and data accuracy, especially over extended periods [36], particularly among older adults [37]. These challenges can result in inaccurate data collection [38]. Nonwearable methods, such as remote monitoring through ambient sensors, offer potential solutions to these issues [37,39,40]. These approaches can alleviate concerns related to device adherence and physical discomfort, providing a more seamless integration into daily life. Additionally, our literature review indicates that most studies on depression classification use a relatively large number of participants and primarily use single-stage classifiers. Many studies also focus on detailed aspects of physical activity data (eg, body displacement, acceleration) using intrusive wearable sensors, which, while effective, present challenges related to user comfort and compliance. This reliance on wearable technology underscores the need for exploring alternative, less intrusive methods.

Recently, Wi-Fi–based sensing in smart homes has emerged as an alternative method for detecting and monitoring contextual human activity and movement [41]. Wi-Fi–based technologies are increasingly adopted due to their existing infrastructure in homes and minimal additional setup costs [42]. These technologies use signal metrics such as received signal strength indicator (RSSI) and channel state information (CSI) to analyze Wi-Fi signal characteristics, offering human activity identification compared to invasive wearable sensors, image analysis, or video-based systems [43]. Leveraging Wi-Fi–based activity data with machine learning presents a viable approach for different diseases, specifically depression classification, which is the focus of this study.

**Figure 1:**
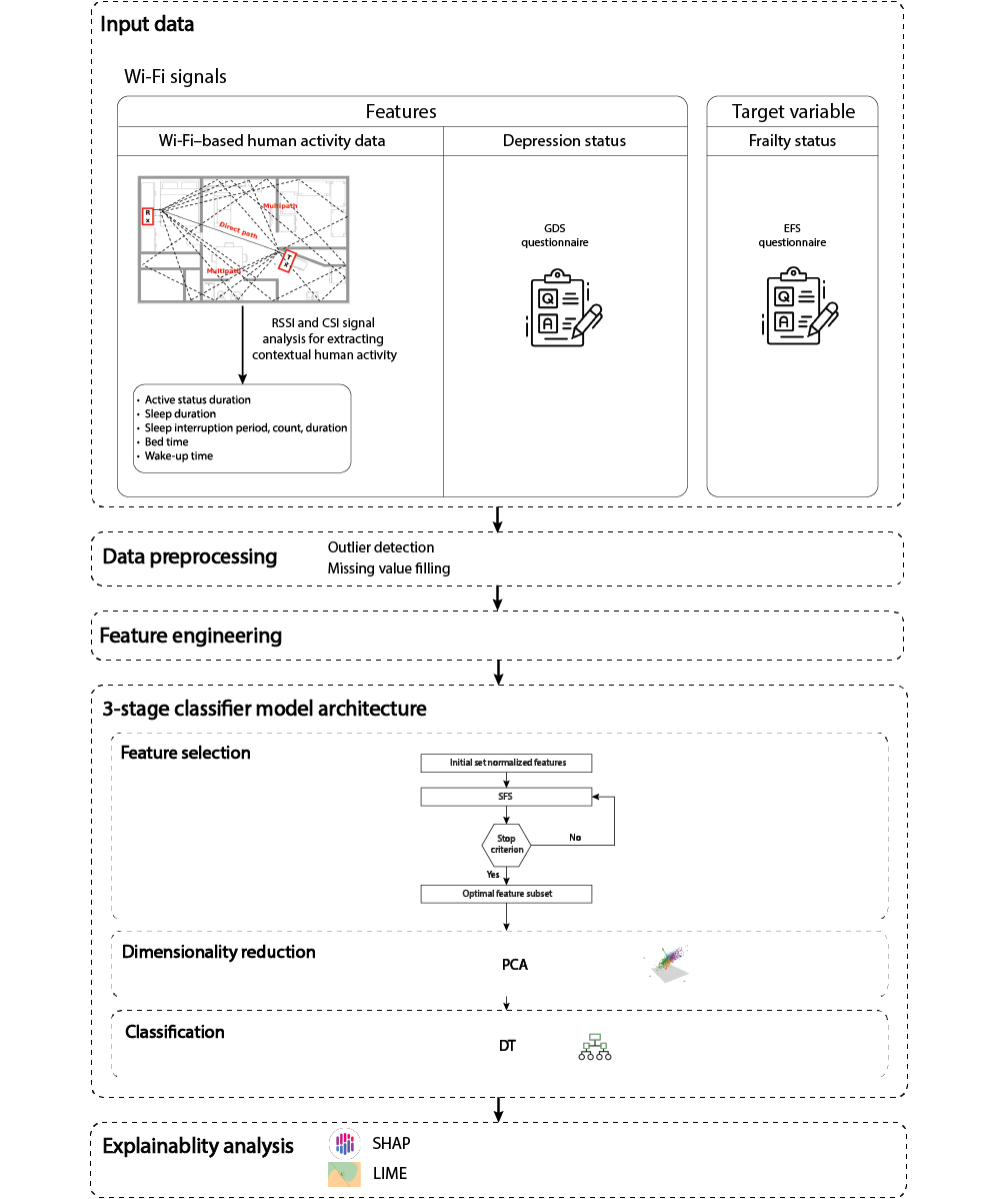
Structure of the automatic Wi-Fi–based depression classification framework. CSI: channel state information; DT: decision tree; EFS: Edmonton Frailty Scale; GDS: Geriatric Depression Scale; LIME: local interpretable model-agnostic explanations; PCA: principal component analysis; RSSI: received signal strength indicator; SFS: sequential forward selection; SHAP: Shapely addictive explanations.

Technological and AI-based methods for depression classification predominantly rely on wearable sensors and are often conducted on large participant groups and typically use statistical techniques or single-stage machine learning classifiers. Furthermore, these approaches often overlook the explainability analysis of the models, which causes a lack of understanding of the underlying decision-making processes and the contribution of individual features to the model’s predictions. To address these challenges, this feasibility study introduces a novel 3-stage machine learning model that incorporates feature selection, dimensionality reduction, and classification for depression classification. This model is specifically designed for a limited number of participants using low cost, easily installable Wi-Fi data, which provides continuous insights into human indoor activities. In addition to creating a model that functions effectively with small sample sizes, this study integrates explainable AI techniques to enhance the interpretability of the model’s predictions. This approach ensures that the insights derived from the model are transparent and comprehensible, providing clarity on how specific features contribute to the classification outcomes. Figure 1 illustrates a schematic overview of our proposed framework.

## Methods

### Data Acquisition

We begin by outlining the data acquisition process for the participants, followed by an in-depth explanation of our proposed HOPE model.

#### Study Cohort

Our recruitment approach used both digital and in-person strategies. Digital outreach was conducted through email campaigns, social media platforms, and digital posters. Prospective participants were provided with detailed information about the study, and those who expressed interest received a consent form. A member of the research team then coordinated the setup of the monitoring equipment. Participants were compensated for their time and involvement with an e-gift card.

Participants in this study were required to be aged 65 years or older, capable of communicating in English or French, and have access to an internet connection at home. Exclusion criteria were as follows: (1) individuals with mental or physical conditions that would impede their ability to participate in the study and its 6-month follow-up, such as gait or balance disorders, active mental health issues, or the use of mobility aids such as canes; and (2) individuals with current substance use disorder, including alcohol or drugs, due to their potential impact on physical mobility. However, individuals with a history of substance use disorder who were no longer consuming were considered eligible.

#### Experimental Protocol

This study used a nonintrusive Wi-Fi–based motion sensor system to facilitate remote monitoring of human activity. Initially, we collected demographic information (ie, age and gender) from the participants. Subsequently, Wi-Fi data were acquired through our remote monitoring technology [44]. This device, installed at the network access point, detects Wi-Fi signals in environments conducive to passive sensing, including private residences and public spaces where Wi-Fi is prevalent [45]. In such indoor settings, Wi-Fi signals exhibit stability in the absence of individuals but fluctuate significantly with the presence and movement of people [45]. These signal variations correspond to distinct patterns associated with human movements and activities, thus providing valuable data for activity monitoring [46]. The collected data were subsequently transferred to secure cloud storage via an internet connection. Our team has developed advanced signal processing and AI-based algorithms to process raw Wi-Fi RSSI and CSI measurements. These algorithms standardize signal variations and translate RSSI and CSI fluctuations into a detailed set of contextual information related to human activity [45]. This information encompasses daily activity duration, bedtime, wake-up time, total sleep duration, and sleep interruption information. In addition to using Wi-Fi–based activity data, frailty and depression statuses were also assessed using validated assessment tools. The Edmonton Frailty Scale (EFS), which measures multiple dimensions of frailty [47-49], and the 15-item Geriatric Depression Scale (GDS) [50] were administered at the end of the experiment.

#### Analytical Framework

The methodical steps of our proposed model are detailed in the following subsections. During the development, implementation, and reporting, we adhered to the Minimum Information About Clinical Artificial Intelligence Modeling (MI-CLAIM) guidelines [51], following best practices designed to promote transparency and reproducibility of our AI model.

#### Data Preparation and Feature Extraction

To prepare the contextual human activity data as input for depression classification, obtained from our Wi-Fi signal analysis software, we designed a preprocessing stage. This process involves handling missing values and outliers to ensure the integrity of the data [52,53]. To enhance analytical depth and improve model performance, we implemented feature engineering on contextual human activity data. This process involves extracting a variety of new features, such as the mean and SD of bedtime and wake-up times, mean and SD of sleep duration (in hours), total count and mean of sleep interruptions, total duration and mean duration of sleep interruptions (in hours), longest continuous sleep duration (in hours), percentage of nights with sleep disturbances, and metrics related to daily activity, including the mean and SD of total daily activity and hourly activity durations, as well as peak activity hour. These derived features, combined with EFS data, were incorporated into our 3-stage machine learning classification model. The depression status, determined using the GDS, was used to label the samples for classification purposes.

#### HOPE Model Development

The proposed HOPE Model was designed for depression classification in older adults using nonintrusive Wi-Fi–based motion sensor data. Due to the limited number of participants, it is necessary to provide an efficient pipeline for preprocessing, feature extraction, and classification. The limited number of participants and high dimensionality of features required a tailored multistage machine learning pipeline to maximize classification accuracy. Furthermore, ensuring that our depression classification model is explainable to clinicians is important, as highlighted in our previous works [54,55]. To address this, we incorporated explainable machine learning techniques such as Shapley additive explanations (SHAP) and local interpretable model-agnostic explanations (LIME). To achieve these goals, our proposed HOPE model was structured into 3 stages of machine learning architecture: feature selection, dimensionality reduction, and classification followed by post hoc explainability analysis using SHAP and LIME. Each stage plays a critical role in refining the data and ensuring that the final classification is both accurate and interpretable. Table 1 provides details on the various techniques used and evaluated at each stage.

**Table 1:** Methods used at each phase of our 3-stage architecture.

Feature selection	Dimensionality reduction	Classification	Explainability analysis
CFS^a^ [56]	PCA^b^ [57]	NB^c^ [58]	SHAP^d^ [59]
SFS^e^ [60]	FA^f^ [61]	LR^g^ [62]	LIME^h^ [63]
MI^i^ [64]	LDA^j^ [65]	kNN^k^ [66]	—^l^
SelectKBest [67]	kPCA^m^ [68]	SVM^n^ [69]	—
RFE^o^ [70]	—	Decision tree [71]	—
—	—	RF^p^ [72]	—
—	—	GBM^q^ [73]	—
—	—	XGboost [74]	—
—	—	LightGBM [75]	—
—	—	Voting classifier [76]	—
—	—	Bagging classifier [77]	—
—	—	AdaBoost [78]	—

^a^CFS: correlation-based selection.

^b^PCA: principal component analysis.

^c^NB: naive Bayes.

^d^SHAP: Shapley addictive explanations.

^e^SFS: sequential forward selection.

^f^FA: factor analysis.

^g^LR: logistic regression.

^h^LIME: local interpretable model-agnostic explanations.

^i^MI: mutual information.

^j^LDA: linear discriminant analysis.

^k^kNN: k-nearest neighbor.

^l^Not applicable.

^m^kPCA: kernel principal component analysis.

^n^SVM: support vector machine.

^o^RFE: recursive feature elimination.

^p^RF: random forest.

^q^GBM: gradient boosting machine.

Feature selection is performed to reduce the dimensionality of the dataset by identifying the most relevant features for depression classification, enhancing both the speed and accuracy of the classification model [79]. The reduced subset of features serves as input for the subsequent dimensionality reduction stage. The validity of the chosen selected features is investigated using correlation analysis in the Results section. Dimensionality reduction techniques are applied to further refine the feature set compared to the initial feature selection stage [80] and to minimize overfitting. The dimensionally reduced features from the second stage were then processed in the third stage, which focused on classification. In this stage, the classification model processes the features derived from the earlier stages to categorize samples into 2 target classes: “participants with depression” and “participants without depression.” The classification algorithm leverages the patterns identified in the features, such as sleep duration and interruptions, to make predictions. The classification task involved assigning a probability score to each sample, determining the likelihood of belonging to either class based on the relationships in the feature set. A decision boundary was then established to assign the final class label for each sample. The classification process was systematically evaluated to ensure robustness and reliability, focusing on separating the 2 groups effectively even with the small dataset. The machine learning classification pipeline was designed to minimize the risk of overfitting by using techniques such as feature selection and dimensionality reduction, ensuring that the most informative and relevant features were used for prediction. To conclude, we used explainable AI techniques to interpret the model’s predictions, focusing on identifying the most influential features and their impact on classification outcomes.

Following the training and evaluation of all potential combinations for each stage, the architecture using sequential forward selection (SFS) for feature selection, principal component analysis (PCA) for dimensionality reduction, and DT for classification emerged as the most effective configuration. This SFS-PCA-DT framework (Figure 1) demonstrated superior performance compared to other combinations.

Our proposed model is supported by different considerations. The SFS algorithm incrementally selects features to improve classification performance and is particularly suited for datasets with a smaller number of participants [81]. Unlike filter methods, SFS acts as a wrapper technique that pairs with a machine learning classification algorithm, providing greater stability in performance [81]. The method starts with no selected features and progressively adds them based on their ability to enhance cross-validation outcomes. In the second stage, PCA serves as a highly effective tool for reducing the dimensionality of data in an unsupervised manner [82]. It converts the initial set of features into a reduced number of uncorrelated components, maintaining the bulk of the data’s variance. This reduction is important for preventing overfitting, especially with limited sample sizes. As a classification algorithm, DT algorithm, known for its strength in binary classification tasks, effectively uses the streamlined feature set generated in earlier stages. It models data by learning simple decision rules inferred from the input features, creating a tree-like structure. Each internal node in the tree represents a decision based on a feature, each branch represents an outcome of the decision, and each leaf node represents a class label [83]. The integration of SFS, PCA, and DT results in an efficient model that aligns with established methodologies and theoretical principles in the field. In this study, comprising only 4 participants, the training and validation procedure were carefully designed to minimize overfitting and to achieve reliable model generalization. To this end, we used a 4-fold cross-validation strategy. Each fold consisted of 3 participants for training and 1 participant for testing, ensuring that every participant contributed to both training and testing in separate iterations. This approach was repeated 10 times with different random seeds to account for variations in the training process, further enhancing the robustness of the performance metrics. During the training phase, a range of hyperparameter optimization techniques, including random search [84], Bayesian optimization [85], and Hyperband [86] was performed for each component of the 3-stage pipeline. For example, the DT classifier’s maximum depth, minimum sample split, and criterion parameters are tuned using Bayesian optimization within predefined search spaces. Similarly, for PCA, the optimal number of components is optimized to maximize variance retention while preventing overfitting. The SFS algorithm was guided by internal cross-validation within the training set to identify the most predictive subset of features.

#### Evaluation Metrics

To validate the effectiveness of our depression classification method, we used 4 evaluation metrics: accuracy [87], sensitivity [87], precision [87], and *F*_1_-score. Accuracy provides a comprehensive measure of the model’s overall performance. Sensitivity helps ensure that the model accurately identifies as many true cases of depression as possible, minimizing the risk of missing individuals who actually have the condition [88]. To further validate the stability of the model, we present the training and test accuracies against the hyperparameter variations, demonstrating the model convergence.

### Ethical Considerations

The study received approval from McGill University’s Institutional Ethics Committee (A06-B18-21A), allowing data collection and analysis for this project. Written informed consent was collected from all participants prior to their involvement in the study. All collected data were anonymized immediately after collection, with no personally identifiable information retained to ensure participant confidentiality. Participants received a $20 e-gift card as compensation for their time. At every stage of the research, we adhered to the ethical principles outlined in the Declaration of Helsinki [89] and the Tri-Council Policy Statement [90].

## Results

### Clinical Study Insights

Six community-dwelling older adults residing in Montreal, Canada, were recruited for this study between May 2022 and September 2022. However, 2 participants withdrew due to internet connectivity issues, resulting in the use of data from the remaining 4 participants for the analysis. The EFS results indicated that 2 participants exhibited moderate frailty (scores ranging from 6 to 11), while the other 2 participants were classified as nonfrail (scores of 5 or below). GDS results suggested that 1 participant had depressive symptoms (score=10), while the other 3 participants did not (score range=0-4).

Some of the participants’ demographic and clinical characteristics are detailed in Table 2. Over a 6-month period, the activities of each participant were continuously monitored at 15-minute intervals using Wi-Fi motion sensors. Following the identification of potential input features derived from Wi-Fi signals and questionnaire data, we designed and developed a 3-stage architecture as outlined in the methodology section. To support future research and ensure the reproducibility of our findings, the code for our model is openly accessible on our lab’s GitHub repository [91].

**Table 2:** Demographic and clinical characteristics of the participants of this study.

	Participant without depression (n=3)	Participant with depression (n=1)
Sex (female/male), n/n	1/2	1/0
Age (years), mean (SD)	67.05 (3.70)	65.50 (0.00)
Edmonton frailty scale, mean (SD)	3.34 (3.21)	7.00 (0.00)
Geriatric depression scale, mean (SD)	4.00 (1.00)	10.00 (0.00)

We assessed a total of 240 model configurations, which resulted from the combination of 5 feature selection methods, 4 dimensionality reduction techniques, and 12 classifiers. Each configuration was trained 10 times to account for variations in performance metrics, ensuring the robustness of our findings. To mitigate the overfitting risk, we used k-fold cross-validation [92]. We experimented with different initial feature sets to optimize model performance, and among the various hyperparameter tuning methods, Bayesian optimization consistently yielded superior results.

### Model Performance and Validation

This section presents the outcomes of our classification model evaluation. Table 3 summarizes the top-performing configurations among the 240 model variations that we tested. The SFS-PCA-DT model, which integrates SFS, PCA, and DT, emerged as the leading performer across multiple metrics. Its relatively high accuracy indicates the model’s ability to distinguish between individuals with and without depression. The model’s high sensitivity ensures that individuals with depression are correctly identified. This is critical in clinical settings, where depression, particularly among older adults, often goes unrecognized despite its severe impact on cognitive function [93-96], quality of life [97,98], and mortality risk [99,100]. Ensuring high sensitivity reduces the likelihood of missed diagnoses, which is important for timely and effective treatment. However, the relatively high standard deviation in both accuracy and sensitivity suggests that the model’s performance may vary, indicating occasional instances of less reliable predictions.

**Table 3:** Average classification performance of the top 5 architectures.

Model	Accuracy (%)	Sensitivity (%)	Precision (%)	*F*_1_-score (%)
SFS^a^ – PCA^b^ – DT^c^	87.50 (12.50)	90.00 (20.00)	88.34 (18.34)	86.00 (14.74)
SFS + FA^d^ + DT	85.00 (16.58)	83.34 (25.82)	90.00 (20.00)	81.67 (18.93)
SFS + PCA + LR^e^	82.50 (19.53)	85.00 (22.91)	83.34 (25.82)	80.00 (20.82)
SFS + PCA + SVM^f^	82.50 (19.53)	90.00 (20.00)	75.00 (22.42)	80.00 (20.82)
MI^g^ + PCA + LR	80.00 (10.00)	85.00 (22.91)	78.34 (22.42)	76.00 (13.06)

^a^SFS: sequential forward selection.

^b^PCA: principal component analysis.

^c^DT: decision tree.

^d^FA: factor analysis.

^e^LR: logistic regression.

^f^SVM: support vector machine.

^g^MI: mutual information.

Table 3 illustrates that SFS is frequently featured among the highest-performing models, including the top model with 87.50% accuracy, and 3 other strong contenders. Mutual information also proves effective, appearing in 1 model with 80.00% accuracy, indicating that both SFS and mutual information are potent feature selection techniques for depression classification with limited samples. PCA seems to be the preferred method for dimensionality reduction, being used in 4 of the top 5 models. Among classifiers, DT stands out, featuring in the top 2 models with 87.50% and 85.00% accuracy. Other classifiers, such as LR and SVM, also perform well, each appearing in models with an accuracy exceeding 80%. The results highlight interesting tradeoffs, such as the SFS + PCA + SVM model, which, while slightly lower in accuracy (82.50%), maintains a high sensitivity (90.00%). This supports the practice of evaluating models using multiple metrics, especially in situations where the application involves clinical diagnoses, where accurately identifying true positives is crucial.

**Figure 2:**
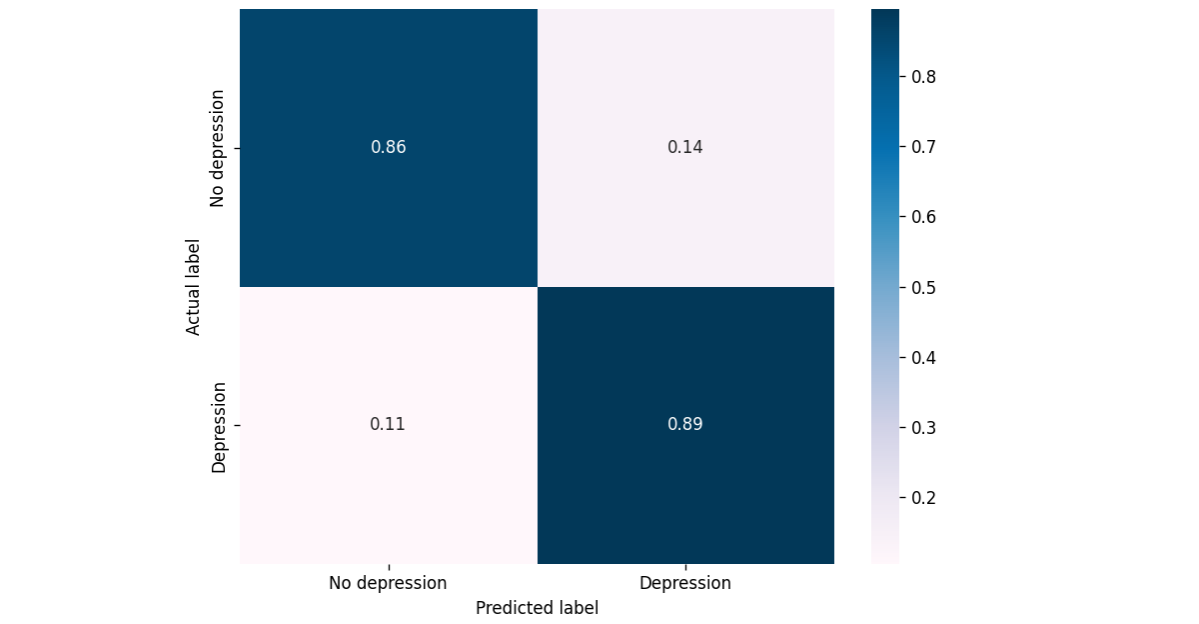
Confusion matrix for the top-performing model (SFS – PCA – DT). DT: decision tree; PCA: principal component analysis; SFS: sequential forward selection.

Figure 2 displays the averaged confusion matrix for the top-performing SFS – PCA – DT model used to classify depression status. Due to the constraint of having only 4 samples, we used a 4-fold cross-validation strategy, with each fold being tested 10 times to ensure a thorough evaluation. The model showed promising results, accurately classifying 86% of individuals without depression as not having depression and 89% of individuals with depression as having depression. The average false positive rate, where individuals without depression were incorrectly classified as having it, was 14%, while the average false negative rate, where individuals with depression were incorrectly classified as not having it, was 11%.

**Figure 3:**
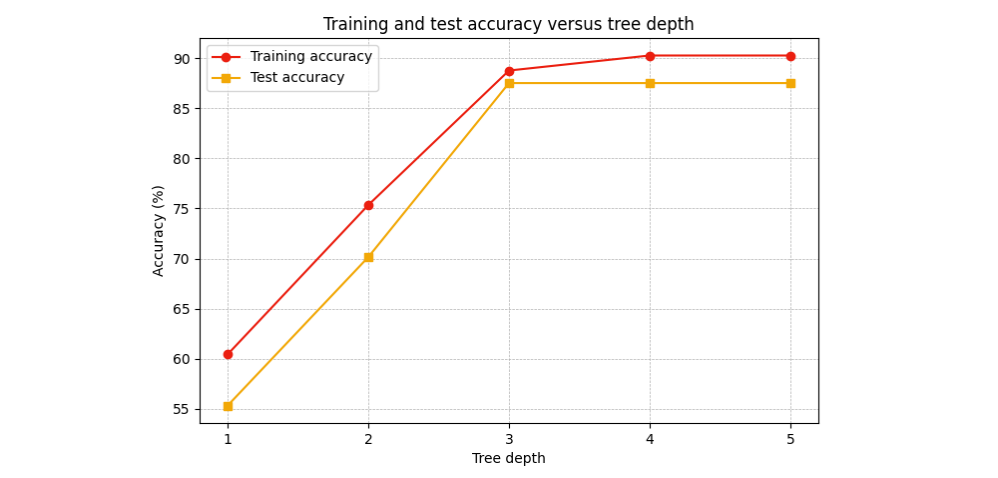
Training and test accuracy as a function of tree depth, demonstrating convergence of our proposed model.

To validate the convergence of the proposed algorithm, we analyzed the relationship between tree depth and accuracy on both the training and test datasets. Figure 3 demonstrates the training and test accuracy of the best performing model (SFS – PCA – DT) as a function of tree depth. The training accuracy increases consistently with tree depth, stabilizing at its maximum, reflecting that the model can fully capture the training data as depth increases. The test accuracy improves initially with increasing tree depth but stabilizes beyond a depth of 3. These observations confirm that the proposed algorithm achieves convergence in terms of performance tradeoffs between model complexity and generalization.

### Feature Selection and Analysis

In our 3-stage classification model, we implement a combination of feature selection and dimensionality reduction techniques to improve the efficacy of our machine learning approach [80]. The features selected by SFS included “average sleep duration,” “total number of sleep interruptions,” “percentage of nights with sleep interruptions,” “average duration of sleep interruptions,” and “EFS.” Correlation analysis of these features revealed notable associations with depression status. Such analysis helps identify how variations in these features might be related to changes in depression, providing valuable insights for clinicians and researchers to develop more effective diagnostic tools and treatments.

**Figure 4:**
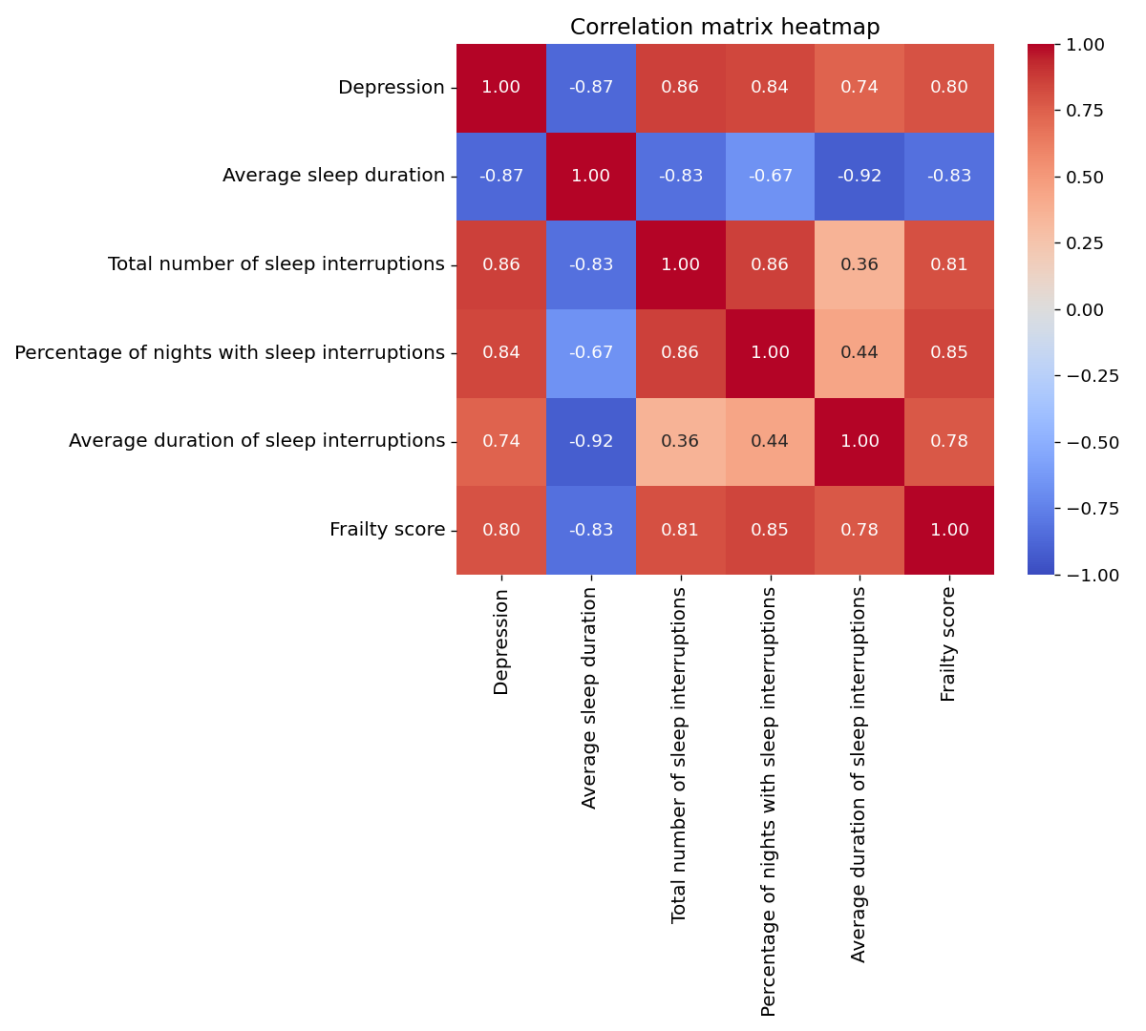
Correlation matrix heatmap between depression and selected features by sequential forward selection.

Figure 4 illustrates a strong negative correlation between depression and average sleep duration. Conversely, depression was positively correlated with the total number of sleep interruptions, percentage of nights with sleep interruptions, average duration of sleep interruptions, and EFS. Blue cells indicate negative correlation values, while red cells represent positive correlations. Darker colors signify stronger correlations. The high correlation values highlight the significance of these factors in understanding and potentially classifying older adults with depression, aligning with findings from previous studies [101-105]. For example, Vallance et al [106] demonstrated that engaging in daily activities can alleviate the adverse effects of depression among older adults. Furthermore, several studies have highlighted a connection between depression and frailty [107,108]. Vaughan et al [109] showed that the prevalence of both depression and frailty among individuals aged 55 years and older exceeds 10%. These findings confirm the association between depression and the features incorporated in our model.

### Comparative Analysis With Baseline Models

In this section, we evaluate the performance of our proposed 3-stage architecture for depression classification, which leverages Wi-Fi–based contextual human activity data, against baseline models previously outlined in the introduction. Although direct comparisons are inherently difficult due to differences in data acquisition methods (wearable devices), feature sets, and sample sizes, this analysis serves to contextualize the effectiveness of our approach for our case study with a limited number of participants.

For the baseline models, we selected the most current classification architectures used in the literature for depression classification. To ensure a fair comparison, each baseline model was trained and tested using the same feature set applied in our experiment. The resulting performance metrics for each baseline model are presented in Table 4. Despite the challenges associated with our smaller sample size, the comparison offers valuable insights into the relative efficacy of our method.

**Table 4:** Average performance across different baseline machine learning models.

Model architecture	Accuracy (%)	Sensitivity (%)	Precision (%)	*F*_1_-score (%)
RF^a^ [23,27]	12.50	5.00	5.00	N/A^b^
SVM^c^ [23]	15.00	10.00	10.00	N/A
LR^d^ [23]	22.50	15.00	13.34	N/A
XGBoost [34]	25.00	10.00	10.00	N/A
L1-based feature selection + DT^e^ [24]	32.50	35.00	18.34	N/A
L1-based feature selection + RF [24]	22.50	15.00	13.34	N/A
L1-based feature selection + kNN^f^ [24]	22.50	15.00	13.34	N/A
L1-based feature selection + NB^g^ [24]	30.00	25.00	15.00	N/A
L1-based feature selection + LR [24]	37.50	45.00	25.00	N/A
L1-based feature selection + SVM [24]	25.00	10.00	10.00	N/A
Randomized LR + AdaBoost [25]	55.00	73.33	55.00	N/A
HOPE model^h^	87.50	90.00	88.34	86.00

^a^RF: random forest.

^b^N/A: data not applicable.

^c^SVM: support vector machine.

^d^LR: logistic regression.

^e^DT: decision tree.

^f^kNN: k-nearest neighbor.

^g^NB: naive Bayes.

^h^Best performed proposed model.

As shown in Table 4, traditional single-stage machine learning classifiers such as RF, LR, and SVM demonstrate relatively lower performance, with accuracy ranging from 12.50% to 22.50%. Among these, LR achieves relatively higher accuracy. XGBoost exhibits better performance than the traditional models. Incorporating feature selection techniques further improves the performance of these models. Specifically, combining L1-based feature selection with various classifiers results in modest performance gains, while the randomized LR combined with AdaBoost achieves a significant improvement, reaching 55.00% accuracy and 73.33% sensitivity. Our proposed 3-stage architectures significantly surpass all other baseline models across all metrics. While most baseline models struggle with sensitivity and precision, often scoring below 15%, our best proposed model demonstrates substantial enhancements in these metrics with 90.00% sensitivity and 88.34% precision, indicating a superior capability to correctly identify positive cases and reduce false positives.

### Model Explainability

To enhance our understanding of the decision-making processes within our proposed model, we used SHAP [50] and LIME [54] for model interpretability analysis. These model-agnostic methods can be applied across various machine learning models, providing valuable insights into our model’s predictive behavior. By integrating these interpretability techniques, we aim to improve the transparency and potential clinical relevance of our depression classification framework. These methods help us identify which features most significantly influence the model’s predictions, particularly in the context of depression classification.

#### SHAP Analysis

**Figure 5:**
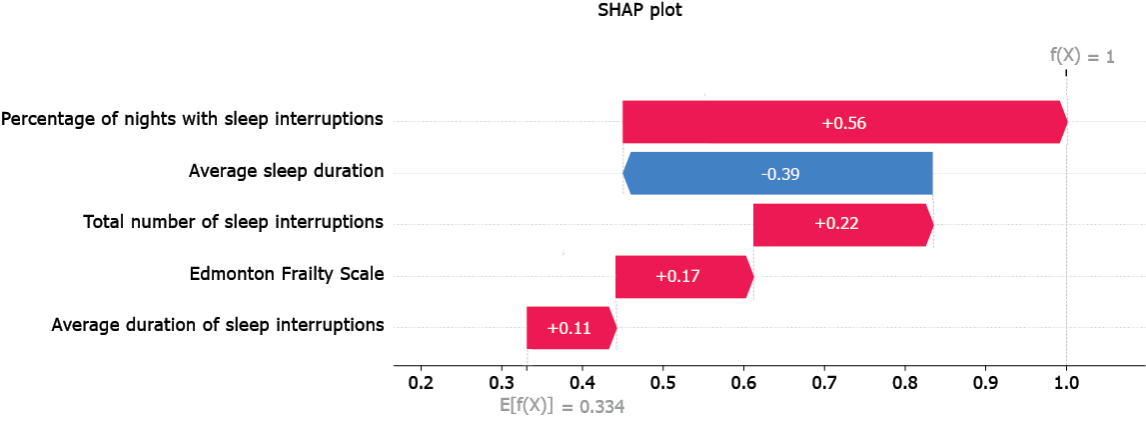
SHAP analysis. SHAP: Shapley additive explanations.

The SHAP waterfall plot (Figure 5) illustrates the relative importance of features for depression classification, with red and blue colors representing positive and negative contributions, respectively. Among the features, “the percentage of nights with sleep interruptions” is the most impactful, positively correlating with depression risk, indicating that frequent sleep disturbances are a strong predictor of depression. Conversely, the average sleep duration exhibits a substantial negative impact on depression prediction, suggesting that longer sleep durations are associated with a reduced likelihood of depression. Sleep-related variables continue to play a pivotal role in the model’s predictions; both the total number of sleep interruptions and the average duration of these interruptions contribute positively to depression risk, further underscoring the importance of uninterrupted sleep in depression diagnosis. Although the frailty scale is included in the model, its influence is relatively minor compared to sleep-related features.

#### LIME Analysis

**Figure 6:**
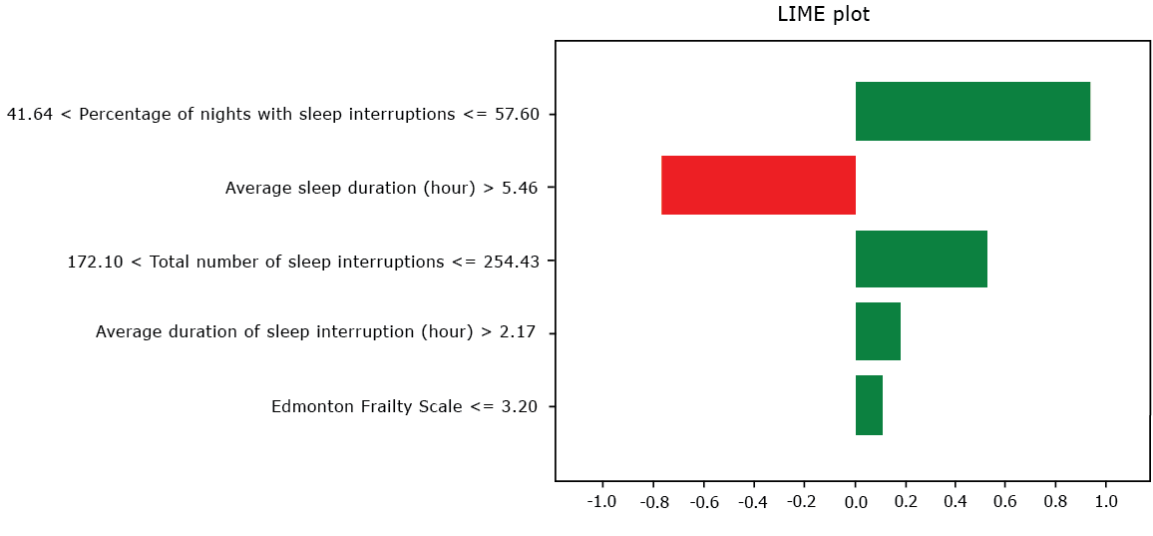
LIME analysis. LIME: local interpretable model-agnostic explanations.

The LIME plot (Figure 6) provides a complementary view of feature importance, with green and red colors indicating positive and negative influences, respectively. Consistent with the SHAP results, LIME identifies “the percentage of nights with sleep interruptions” as the most critical feature in the classification of depression of our proposed model. Similarly, the average sleep duration is shown to have a significant negative impact on depression classification, in line with SHAP findings. The total number of sleep interruptions also ranks highly with a positive influence on depression risk, again aligning with SHAP results. A notable difference between the 2 methods is the relatively lower impact of the frailty scale in the LIME analysis, which requires further investigations.

## Discussion

Our research uses Wi-Fi–based motion sensors to extract daily activities, which are then used in our proposed machine learning method for depression classification.

Our study findings confirm the feasibility of using Wi-Fi–based motion sensors for depression classification among older adults. Our proposed HOPE (Home-Based Older Adults’ Depression Prediction) model achieved an accuracy of 87.5%, sensitivity of 90%, and precision of 88.3%. The most influential features identified were sleep-related metrics, such as average sleep duration and sleep interruptions, highlighting the importance of sleep patterns in depression classification. These findings suggest that Wi-Fi–based monitoring offers a nonintrusive and effective alternative to conventional wearable technologies for depression assessment. These conventional methods, while effective, often present challenges in terms of participant compliance, particularly among older adults, due to their burdensome and sometimes uncomfortable nature. In contrast, our Wi-Fi–based approach is nonintrusive and allows for continuous monitoring without requiring participants to wear or interact with any devices. This can significantly enhance participant compliance and the integrity of the data collected over extended periods. Compared to other nonintrusive monitoring technologies, such as camera-based methods, our Wi-Fi–based approach has distinct advantages. Wi-Fi infrastructure is prevalent in most homes and does not pose privacy risks, making it a cost-effective and scalable solution for continuous health monitoring. Furthermore, unlike previous studies that rely on microlevel body displacement and accelerometer data, our study emphasizes macrolevel physical activity features like sleep patterns and overall activity levels shift is crucial as it highlights the potential of using broader, more easily obtainable metrics to assess depression status. Our findings demonstrate that these macrolevel features are not only feasible but also effective measures for depression classification, broadening the scope of nonintrusive monitoring technologies in mental health research. The next steps can be extracting more detailed types of human activity using nonintrusive Wi-Fi data and expanding more on using this type of data acquisition for depression classification.

Additionally, our proposed model demonstrates relatively high performance compared to other classification models presented in existing depression classification studies, even with a limited sample size. Many studies using physical activity data from wearable devices often benefit from larger datasets and frequently use single-stage classifiers or deep neural networks. These models generally show strong performance with abundant data; however, their effectiveness diminishes when applied to smaller datasets, such as the one in this feasibility study. To address the limitations imposed by our smaller sample size, we designed a 3-stage machine learning classification architecture, which combines feature selection, dimensionality reduction, and classification into a multistep process. This approach allows for the extraction of the most relevant features while minimizing noise, thereby improving classification performance. Despite the small sample size, our model consistently outperformed conventional single-stage classifiers, highlighting the strength of both the machine learning architecture and the selected human activity features—particularly sleep patterns and activity levels—used for depression classification. This also underscores the adaptability of our model to different data scales, making it a more versatile option for future research where data availability might be limited. While this model shows promising results, however, caution is needed in interpreting these results. Future work should aim to enhance its robustness and generalizability by expanding the dataset. Collecting Wi-Fi–based physical activity data from a larger and more diverse sample would not only improve the model’s statistical power but also allow for a more comprehensive evaluation of its performance across different population groups, such as varying age ranges and health conditions. This would be particularly valuable in developing a scalable solution for real-world applications. Additionally, the integration of advanced machine learning techniques, such as deep neural networks or hybrid models combining traditional classifiers with deep learning components, could further enhance classification accuracy.

Our study is distinctive not only in its methodological approach but also in its emphasis on model explainability, a crucial aspect often overlooked in prior research on depression classification. Explainability is essential in health care applications, where understanding the factors driving a model’s decision is critical for clinical adoption and trust. By using SHAP and LIME, we were able to dissect the decision-making process of our model and pinpoint the most influential features for classifying depression. Both explainability analyses converge on the identification of sleep interruption features as key predictors in the depression classification of our proposed model. Among these, the “percentage of nights with sleep interruptions,” “average sleep duration,” and “total number of sleep interruptions” emerged as the primary driving factors. These findings align with existing literature that highlights the strong correlation between sleep disturbances and depression. However, our approach goes a step further by quantifying the impact of these features on the classification outcomes, providing a more nuanced understanding of their role. These findings suggest that future tools for depression assessment may benefit from a stronger focus on sleep quality and patterns and further investigations are required in this regard.

The integration of sensors and AI is transforming health care, yet the application of these technologies in depression classification remains underdeveloped and lacks extensive investigation. This study aimed to create an automated machine learning system for the continuous, remote monitoring and assessment of daily physical activity among older adults in a home setting, with the goal of distinguishing between individuals with and without depression.

In summary, although there were some challenges, our results suggest that using Wi-Fi–based data to capture contextual human activities is a promising and efficient method for classifying depression. The model we developed, leveraging data from Wi-Fi motion sensors, showed strong potential in accurately identifying early signs of depression and paving the way for more advanced and accessible mental health monitoring technologies among community dwelling older adults..

## Abbreviations

AI: artificial intelligence
CNN: convolutional neural network
CSI: channel state information
DT: decision tree
EFS: Edmonton Frailty Scale
GDS: Geriatric Depression Scale
LIME: local interpretable model-agnostic explanations
LR: logistic regression
MI-CLAIM: Minimum Information About Clinical Artificial Intelligence Modeling
PCA: principal component analysis
RF: random forest
RSSI: received signal strength indicator
SFS: sequential forward selection
SHAP: Shapely addictive explanations
SVM: support vector machine

## Data Availability

The data analyzed during this study are not publicly available due to restrictions placed by the McGill University Institutional Ethics Committee, but the deidentified data are available from the corresponding author on reasonable request.
